# Terra incognita—cerebellar contributions to neuropsychiatric and cognitive dysfunction in behavioral variant frontotemporal dementia

**DOI:** 10.3389/fnagi.2015.00121

**Published:** 2015-07-02

**Authors:** Rachel H. Tan, Emma Devenney, Matthew C. Kiernan, Glenda M. Halliday, John R. Hodges, Michael Hornberger

**Affiliations:** ^1^Ageing and Neurodegeneration, Neuroscience Research AustraliaRandwick, NSW, Australia; ^2^School of Medical Sciences, University of New South WalesSydney, NSW, Australia; ^3^Sydney Medical School, Brain and Mind Research Institute, University of SydneySydney, NSW, Australia; ^4^Australian Research Council Centre of Excellence in Cognition and Its DisordersSydney, NSW, Australia; ^5^Department of Clinical Neurosciences, Cambridge UniversityCambridge, UK

**Keywords:** cerebellum, behavioral variant frontotemporal dementia, cognition, neuropsychiatric processes, neural correlates

## Abstract

Although converging evidence has positioned the human cerebellum as an important relay for intact cognitive and neuropsychiatric processing, changes in this large structure remain mostly overlooked in behavioral variant frontotemporal dementia (bvFTD), a disease which is characterized by cognitive and neuropsychiatric deficits. The present study assessed whether degeneration in specific cerebellar subregions associate with indices of cognition and neuropsychiatric performance in bvFTD. Our results demonstrate a relationship between cognitive and neuropsychiatric decline across various domains of memory, language, emotion, executive, visuospatial function, and motivation and the degree of gray matter degeneration in cerebellar lobules V–VII. Most notably, bilateral cerebellar lobule VII and the posterior vermis emerged as distinct for memory processes, the right cerebellar hemisphere underpinned emotion, and the posterior vermis was highlighted in language dysfunction in bvFTD. Based on cortico-cerebellar connectivity maps, these findings in the cerebellum are consistent with the neural connections with the cortices involved in these domains in patients with bvFTD. Overall, the present study underscores the significance of cortical-cerebellar networks associated with cognition and neuropsychiatric dysfunction in bvFTD.

## Introduction

The human cerebellum has long been regarded as an important relay station for intact motor function but converging evidence has now established its significant involvement also in cognitive and neuropsychiatric processes (Krienen and Buckner, 2009; O'Reilly et al., 2010; Stoodley and Schmahmann, 2010). It is perhaps of some surprise then that changes in this large brain region remain mostly overlooked in behavioral variant frontotemporal dementia (bvFTD), a disease characterized by neuropsychiatric and cognitive deficits, and that demonstrates motor features of amyolateral sclerosis (ALS) in a subpopulation of patients (Bak and Hodges, 2001). Regardless of the presence of concomitant ALS (Lillo et al., 2010), the progressive deterioration in behavior and personality in bvFTD (Rascovsky et al., 2011) has been largely attributed to degeneration in the prefrontal, insular and temporal cortices (Rabinovici et al., 2007; Seeley et al., 2008). These cortical regions are known to demonstrate dense reciprocal connections with the cerebellum (Middleton and Strick, 2000, 2001; O'Reilly et al., 2010) and we recently confirmed that the degeneration of particular cerebellar subregions impacts on the overall cognitive and neuropsychiatric performances in bvFTD (Tan et al., 2014). However, the involvement of cerebellar substructures in specific cognitive and neuropsychiatric processes in bvFTD has yet to be examined.

The present study set out to extend on our previous findings of cerebellar contributions to global cognitive and neuropsychiatric measures, and examine the involvement of cerebellar subregions to more specific indices of cognition (memory, language, executive, emotion, visuospatial function) and neuropsychiatric performance (abnormal behavior, motivation, stereotypic behavior, mood, eating habits, and beliefs). A functional topographical map has been proposed in the human cerebellum, with lobules I–V and VIII found to be involved in sensorimotor function, contralateral lobules VI and VII in cognitive processing, and the posterior vermis conveying the limbic cerebellum (Krienen and Buckner, 2009; O'Reilly et al., 2010; Stoodley and Schmahmann, 2010; Stoodley et al., 2012). Based on these connectivity studies as well as the neural correlates of cognitive and neuropsychiatric domains identified in the cortices (Grossman et al., 2004; Huey et al., 2009; Raczka et al., 2010; Pennington et al., 2011; Kumfor et al., 2013; Woost et al., 2013; Irish et al., 2014) we predicted significant involvement of cerebellar lobules VI and VII across neuropsychiatric and cognitive domains with greater right lateralized involvement in executive function and language, and left lateralized involvement in visuospatial function in bvFTD.

## Methods

### Case selection

A total of 53 participants took part in this study. Patients were sourced from the FTD Research Clinic, FRONTIER, resulting in a sample of 23 bvFTD, 15 ALSFTD, and 15 controls. All FTD patients met current consensus criteria for bvFTD (Rascovsky et al., 2011), showing the progressive behavioral and/or cognitive decline characteristic of this dementia subset, including some to all of the following: disinhibition, apathy, inertia, loss of empathy, perseveration, stereotypic behaviors and dysexecutive syndrome. Patients also met criteria of evidence of atrophy localized to anterior and/or temporal lobes via MRI. The ALSFTD group comprised patients who met diagnostic criteria for both bvFTD and ALS according with El Escorial Criteria Revised (Brooks et al., 2000), showing both upper and lower motor neuron signs and progressive behavioral/cognitive dysfunction. Well-matched healthy controls were recruited from the Frontier database. Testing and scanning was conducted at the first clinic visit of each patient. Only two bvFTD patients of the included cohorts had the C9orf72 mutation.

### Ethics statement

Ethics approval was obtained from the Human Research Ethics Committee of South Eastern Sydney/Illawarra Area Health Service (HREC 10/126). Research was conducted following the ethos of the Declaration of Helsinki. Written consent, either from patient or family, was obtained for each participant in the study.

### Cognitive and neuropsychiatric assessments

All participants were assessed on the Addenbrooke's Cognitive Examination Revised (ACE-R) (Mioshi et al., 2006) as a measure of general cognitive ability. Specific cognitive assessments were performed as follows: The Rey Auditory Verbal Learning Test (RAVLT) was administered as an index of verbal recall and recognition, the Rey-Osterrieth Complex Figure Test (RCF) was used to assess visual recall, and the Doors and People test (part A) a measure of visual recognition. These three tasks have been described in greater detail previously (Pennington et al., 2011). The Boston Naming Test (BNT) (Goodglass, 2000) and Sydney Language Battery (SYDBAT) (Savage et al., 2013) were administered as standardized indices of verbal semantic performance. The Digit Span Reverse and FAS Verbal Fluency tests were used as measures of executive performance as described previously (Hornberger et al., 2008). Participants were given the Ekman 60 Faces Test obtained from the Facial Expressions of Emotion—Stimuli and Tests (FEEST) (Young Aw et al., 2002) and the facial emotion recognition task, which comprises the facial perceptual task (FPT), facial identity discrimination task (FIDT), facial affect discrimination task (FADT), and facial affect selection task (FAST) as previously described (Miller et al., 2012; Kumfor et al., 2014). Behavioral disturbances in the patients were assessed via the Cambridge Behavioral Inventory (CBI), which is an 81 item questionnaire that assesses cognitive, behavioral and affective symptoms as well as activities of daily living and evaluates various functional/behavioral domains using a 5 point rating scale (Wedderburn et al., 2008). The following scores of the CBI-R were evaluated and analyzed: abnormal behavior, motivation, stereotypic behavior, mood, eating habits, and beliefs. For all assessments, only data collected at the time of neuroimaging scans were included in the analyses.

### Composite scores

All neuropsychological test scores were converted into percentage of the control mean, before averaging to yield composite scores. Scores for the RAVLT, RCF, and Doors and People test (part A) were averaged to produce a memory composite. Scores for the BNT and SYDBAT were averaged to produce a language composite. Composite scores for executive function were derived from the Digit Span Reverse and FAS Verbal Fluency test scores, and scores for emotion derived from the Ekman 60 Faces Test and emotion selection task.

### Statistics

Data were analyzed using IBM SPSS 20.0. A priori, variables were plotted and checked for normality of distribution by Kolmogorov-Smirnov tests. Parametric demographic data (age, education), cognitive (ACE-R) and neuropsychiatric (CBI-R) data were compared across the three groups (bvFTD, ALS-FTD, and controls) via One-Way ANOVAs followed by Tukey HSD *post-hoc* tests. Variables revealing non-normal distributions were log transformed and the appropriate log values were used in the analyses, but Table 1 reports their original values to facilitate clinical interpretation. Variables showing non-parametric distribution after log transformation were analyzed via Chi-square (gender) and Kruskal-Wallis & Mann-Whitney U (disease duration) tests.

**Table 1:** **Demographics, cognition, and neuropsychiatric measures in ALSFTD, bvFTD, and control groups**.

	**ALSFTD**	**bvFTD**	**Controls**
	**(*n* = 15)**	**(*n* = 23)**	**(*n* = 15)**
**DEMOGRAPHICS AND GENERAL COGNITION**			
Age (y)	63 ± 7.6	62 ± 10.1	63 ± 4.9
Education (y)	13 ± 3.6	12 ± 3.1	14 ± 1.7
Gender (M/F)	10/5	15/8	7/8
Disease duration (y)	3 ± 2.3	4 ± 2.4	N/A
ACE-R (total score: 0–100)	63 ± 38^**^	74 ± 31^**^	96 ± 43
**EXECUTIVE**			
Composite score	30.2 ± 22.9^**^	52.1 ± 25.7^**^	100 ± 21.0
DigitSpan backwards	2.9 ± 2.5^**^	4.4 ± 2.5^**^	8.6 ± 2.5
Letter fluency	13.1 ± 11.6^**^	21.8 ± 11.6^**^	45 ± 12.8
**LANGUAGE**			
Composite score	67.1 ± 25.9^*^	80.3 ± 27.2^*^	99.9 ± 3.5
Boston naming	9.5 ± 3.3^*^	11.9 ± 2.8^*^	14.5 ± 0.8
Sydney battery			
Naming	14.6 ± 17.6^*^	19.0 ± 6.5^*^	27.4 ± 1.8
Comprehension	21.6 ± 6.3^*^	24.0 ± 5.4^**^	29.5 ± 0.8
Semantic	19.6 ± 8.9^*^	21.2 ± 6.3^*^	28.4 ± 1.1
**MEMORY**			
Composite score	48.3 ± 32.0^**^	53.7 ± 25.7^**^	100 ± 16.5
RCF 3 min	7.0 ± 8.2	8.5 ± 6.8^*^	17.8 ± 4.8
RAVLTA6	4.1 ± 4.3^*^	4.0 ± 2.9^**^	9.6 ± 2.9
Doors	8.8 ± 2.7	7.7 ± 2.8^**^	11.4 ± 0.7
**VISUOSPATIAL**			
ACE-R (visuospatial: 0–100)	79 ± 17^*^	86 ± 15^*^	98 ± 4
**EMOTION**			
Composite score	71.7 ± 18.6^**^	73.2 ± 16.5^**^	100 ± 3.7
Ekman60	33.0 ± 14.2	35.6 ± 8.1	50.9 ± 4.5
FPT	30.7 ± 8.1	32.8 ± 8.9^*^	39.2 ± 1.4
FIDT	26.0 ± 5.0^*^	26.3 ± 6.4^**^	36.1 ± 4.3
FAST	22.2 ± 9.0^*^	26.9 ± 9.2^**^	39.0 ± 1.7
FADT	26.3 ± 5.5^*^	28.5 ± 6.8^*^	36.9 ± 1.5
**CBI**			
Abnormal behavior (0–100)	25.3 ± 5.9^*^	36.1 ± 4.8^**^	1.1 ± 6.6
Motivation (0–100)	36.1 ± 8.3^*^^,^^+^	66.7 ± 6.8^**^	2.3 ± 9.3
Stereotypic behavior (0–100)	45.1 ± 6.8^*^	54.2 ± 5.6^**^	6.3 ± 7.7
Mood (0–100)	21.0 ± 5.3	34.2 ± 4.4^*^	5.5 ± 6.0
Eating (0–100)	29.5 ± 5.9	47.6 ± 4.8^**^	7.4 ± 6.7
Everyday skills (0–100)	19.3 ± 5.8	30.5 ± 4.7^*^	0.5 ± 6.5
Beliefs (0–100)	1.8 ± 4.1	9.9 ± 3.3	0.0 ± 4.6

Data are presented as mean ± standard deviation. Composite scores are presented as a % of mean control. Differences between groups are represented as

* p < 0.05 compared to controls;

** p < 0.001 compared to controls;

+ *p < 0.05 compared to bvFTD. FPT, facial perceptual task; FIDT, facial identity discrimination task; FADT, facial affect discrimination task; FAST, facial affect selection task*.

### Imaging acquisition and voxel-based morphometry (VBM) analysis

Subjects were scanned using a 3T Philips MRI scanner. T1-weighted acquisition: coronal orientation, matrix 256 × 256 × 200, 161 mm^2^ in-plane resolution, slice thickness 1 mm, *TE*/*TI* = 2.6/5.8 ms.

Voxel-based morphometry (VBM) was conducted on the three dimensional T1-weighted scans, using the FLS-VBM toolbox in the FMRIB software library package (http://www.fmrib.ox.ac.uk/fsl/). The first step involved extracting the brain from all scans using the BET algorithm in FSL, using a fractional intensity threshold of 0.22 (Smith, 2002). Each scan was visually checked after brain extraction, both to ensure that no brain matter was excluded, and no non-brain matter was included (e.g., skull, optic nerve, dura mater).

If non-brain matter was visually detected or brain matter was falsely excluded, the BET algorithm for that scan was repeated with a modified fractional intensity threshold, to give smaller or larger brain border estimates. A gray matter template, specific to this study, was then built from canvassing 10 scans from each group (total *n* = 30). An equal amount of scans across groups was used to ensure equal representation, and thus avoid potential bias toward any single group's topography during registration. Template scans were then registered to the Montreal Neurological Institute Standard space (MNI 152) using non-linear b-spline representation of the registration warp field, resulting in study-specific gray matter template at 2 × 2 × 2 mm^3^ resolution in standard space. Simultaneously, brain-extracted scans were also processed with the FMRIB's Automatic Segmentation Tool (FAST v4.0) (Zhang et al., 2001) to achieve tissue segmentation into CSF, gray matter, and white matter. Specifically this was done via a hidden Markov random field model and an associated Expectation-Maximization algorithm. The FAST algorithm also corrected for spatial intensity variations such as bias field or radio-frequency inhomogeneities in the scans, resulting in partial volume maps of the scans. The following step saw gray matter partial volume maps then non-linearly registered to the study-specific template via non-lia b-spline representation of the registration warp. These maps were then modulated by dividing by the Jacobian of the warp field, to correct for any contraction/enlargement caused by the non-linear component of the transformation (Andersson et al., 2007a). After normalization and modulation, smoothing the gray matter maps occurred using an isotropic Gaussian kernel (standard deviation = 3 mm; full width half maximum = 8 mm). The statistical analysis was performed with a voxel-wise general linear model after correcting for intracranial volume. Significant clusters were formed by employing the threshold-free cluster enhancement (TFCE) method (Smith and Nichols, 2009). The TFCE method is a cluster-based thresholding method which does not require the setting of an arbitrary cluster forming threshold (e.g., t, z). Instead, it takes a raw statistics image and produces an output image in which the voxel-wise values represent the amount of cluster-like local spatial support. The TFCE image is then turned into voxel-wise *p*-values via permutation testing. We employed a permutation-based non-parametric testing with 5000 permutations (Nichols and Holmes, 2002).

### Region-of-interest mask

A region-of-interest (ROI) mask was created for subregions of the cerebellum using the Schmahmann probabilistic atlas of the human cerebellum (http://www.icn.ucl.ac.uk/motorcontrol/imaging/propatlas.htm) as previously described (Tan et al., 2014).

### VBM: correlations with test scores

In a first step, correlations between performance on cognitive and neuropsychiatric assessments with regions of gray matter atrophy in the cerebellum were investigated across participant groups by entering the total scores of ACE-R and CBI-R, and the composite scores of language, memory, emotion, executive and visuospatial assessments as a covariate in the design matrix of the VBM analysis of the cerebellum across participant groups. For statistical power, a covariate only statistical model with a (1) t-contrast was used, providing an index of association between gray matter intensity and clinical scores. In a second analysis, we performed an inclusive masking analysis to verify which areas of cerebellar atrophy that correlate with language, memory, emotion, executive, and visuospatial scores would overlap across cognitive domains. In a final step, we performed an exclusive masking analysis to determine which areas of cerebellar atrophy were specific to language, memory, emotion, executive, and visuospatial scores.

Anatomical locations of significant results were overlaid on the MNI standard cerebellum image for spatial normalization and visual comparison with a brain atlas, allowing localization of areas of significant gray matter loss. The covariate analyses were tested for significance at *p* < 0.01 uncorrected with a voxel cluster threshold of 20 contiguous voxels. Given that cerebellar volumes in these same patient cohorts had already been contrasted previously Supplementary Figure 1 (Tan et al., 2014), group differences were not assessed in the present study.

## Results

### Demographics

Demographic and clinical information is presented in Table 1. The participant groups were well-matched for age (*p* = 0.66), years in education (*p* = 0.32), gender (*p* = 0.78), and disease duration (i.e., the number of months elapsed between onset of symptoms and cognitive testing, *p* = 0.88).

### Test scores

Table 1 details cognitive and neuropsychiatric assessments as well as composite scores for each task category across participant groups. Across the various cognitive and neuropsychiatric assessments administered, a significant poorer performance was seen in both patient groups in comparison to controls (Table 1). No significant difference was identified between patient cohorts with the exception of the motivation component of the CBI-R assessment, where a poorer performance was seen in patients with bvFTD (*p* < 0.05).

### Neural correlates of cognitive and neuropsychiatric scores

**Figure 1:**
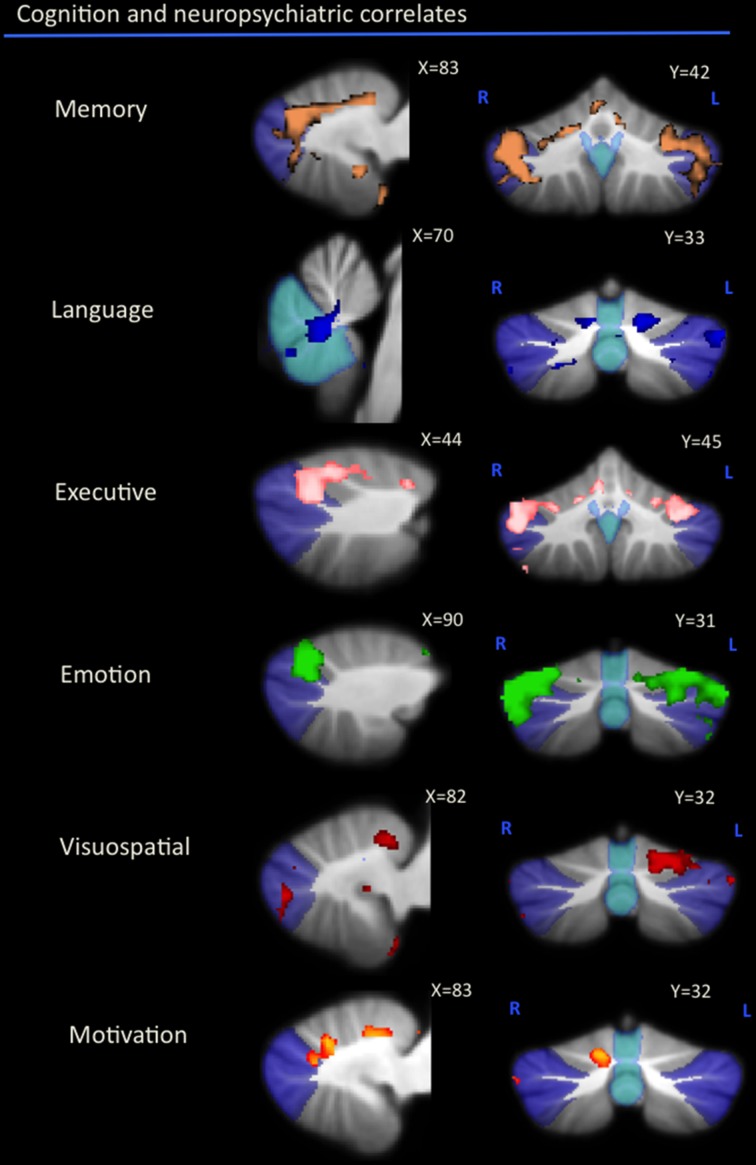
**Voxel-based morphometry analyses showing cerebellar regions in which gray matter intensity correlates significantly with memory, language, executive, emotion, visuospatial task performance, and motivation measures across all participant groups**. Colored voxels show regions that were significant in the analyses for *p* < 0.01 uncorrected and a cluster threshold of 20 contiguous voxels. All clusters reported *t* > 3.5. Clusters are overlaid on the MNI standard brain with a mask for lobule VII (crus 1, 2, and VIIb) shown in blue and a mask for the vermis shown in light blue. L, Left Hemisphere; R, Right Hemisphere.

As can be seen in Figure 1 and Table 2, correlations between cerebellar gray matter atrophy with cognitive and neuropsychiatric measures were examined across all groups. Memory scores (brown color, Figure 1 and Table 2) correlated significantly with gray matter volumes in the posterior lobules VI–VIIB and vermis, with mild involvement identified in anterior lobules I–V. Composite scores of language (blue color, Figure 1 and Table 2) correlated significantly with gray matter volumes in the posterior lobule VI, left lobule VII (Crus I) and the vermis, with mild involvement also seen in lobule V. Executive measures (pink color, Figure 1 and Table 2) were found to associate with gray matter volumes in the posterior lobule VI and VII (Crus 1), with a mild association seen in lobule V. Emotion scores (green color, Figure 1 and Table 2) correlated with gray matter atrophy in the posterior lobule VI and Crus I. Visuospatial measures (red color, Figure 1 and Table 2) correlated with gray matter volume in the left cerebellar lobule VI. Patients' performance on tasks of motivation (red-yellow color, Figure 1 and Table 2) showed a significant association with gray matter volume in right-lateralized lobule VI. No significant correlations were identified between cerebellar volumes with neuropsychiatric measures of abnormal behavior, motivation, stereotypic behavior, mood, eating habits, and beliefs.

**Table 2:** **Voxel-based morphometry (VBM) findings demonstrating gray matter volumes in cerebellum subregions showing a significant correlation with measures of memory, language, executive, emotion, visuospatial task performances (*p* < 0.01) shown in Figure 1**.

**Cerebellar subregions**	**Memory**	**Language**	**Executive**	**Emotion**	**Visuospatial**
I–IV	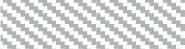				
V	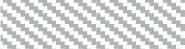	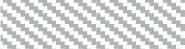	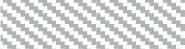		
VI					Left
VII (Crus 1)		Left			
VII (Crus 2)					
VIIB					
VIII					
IX–X					
Vermis					

*Solid gray color represents areas of substantial correlation, diagonal lines represent areas of milder significance*.

### Overlap analysis

**Figure 2:**
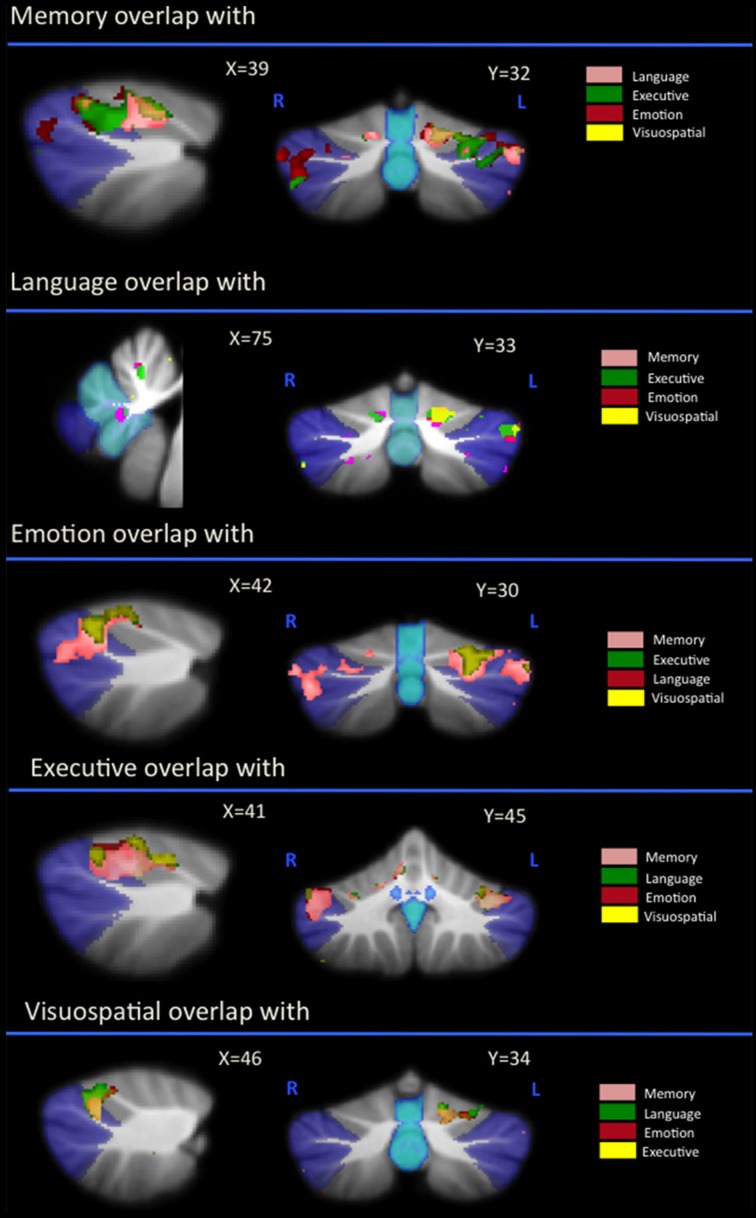
**Voxel-based morphometry analyses showing overlapping regions of cerebellar gray matter atrophy for memory, language, emotion, executive, and visuospatial performances across all participants**. Colored voxels show regions that were significant in the analyses for *p* < 0.01 uncorrected and a cluster threshold of 20 contiguous voxels. All clusters reported *t* > 3.5. Clusters are overlaid on the MNI standard brain with a mask for lobule VII (crus 1, 2, and VIIb) shown in blue and a mask for the vermis shown in light blue. L, Left Hemisphere; R, Right Hemisphere.

Regional gray matter in the cerebellum associated with memory, language, executive, emotion, and visuospatial scores were further investigated to determine regions of overlap with other cognitive domains. Table 3 and Figure 2 demonstrates significant overlap across all cognitive domains in the left cerebellar lobule VI, with further shared representations seen in the right cerebellar lobule VI and bilateral lobules V and VII (Crus I) for memory, language, executive and emotion processes. A further shared representation was seen in the vermis shared between memory and language domains.

**Table 3:** **Voxel-based morphometry (VBM) findings demonstrating shared regions of cerebellar gray matter atrophy for memory (brown color), language (blue color), emotion (green color), executive (pink color), and visuospatial performances (red color) across all participants (*p* < 0.01)**.

Cerebellar subregions	Memory					Language						Executive					Emotion					Visuospatial											
**I-IV**																																	
**V**	R L	R L				R L		R L				R L	R L				R L	R L															
**VI**	R L	R L	L	L		L	L	L	L			R L	R L	L	L		R L	R L	L	L		L	L	L	L								
**VII (Crus 1)**	R L	R L	L			L	L	L				R L	R L	L			R L	R L	L														
**VII (Crus 2)**																																	
**VIIB**																																	
**VII X**																																	
**Vermis**	R L					R L																											

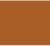
 Memory

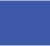
 Language

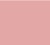
 Executive

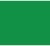
 Emotion

*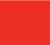 Visuospatial*.

### Difference analysis

**Figure 3:**
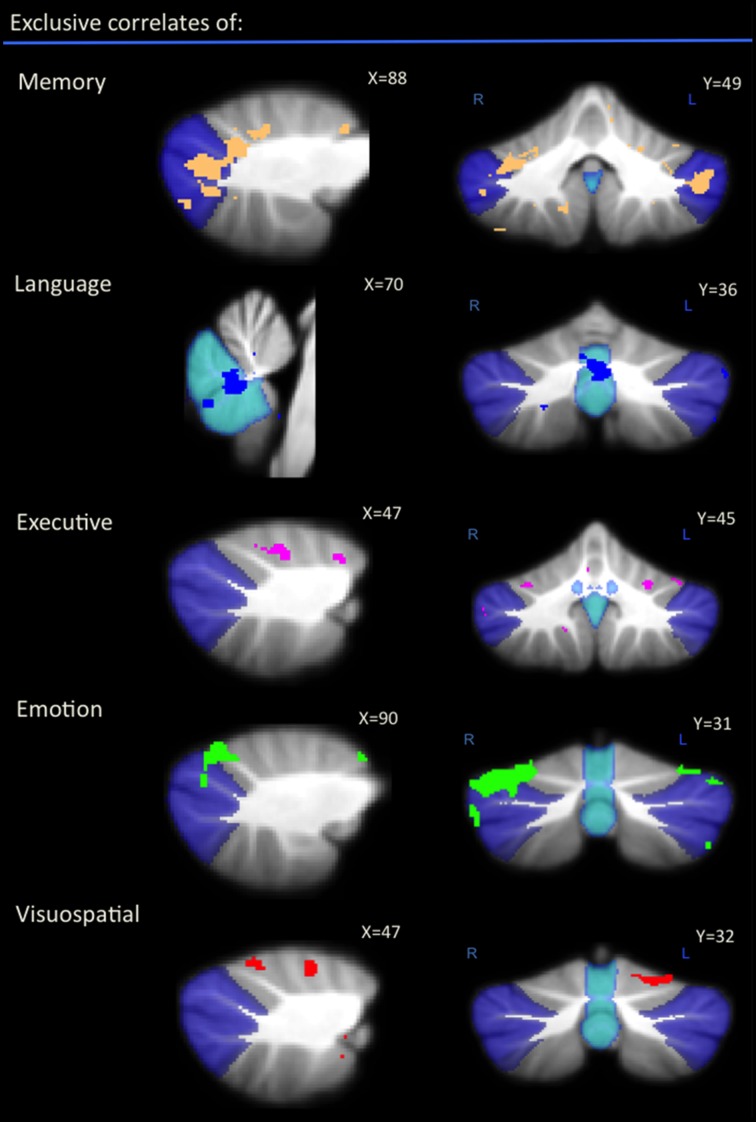
**Voxel-based morphometry analyses showing exclusive regions of cerebellar gray matter correlates for memory, language, emotion, executive, and visuospatial performances across all participants**. Colored voxels show regions that were significant in the analyses for *p* < 0.01 uncorrected and a cluster threshold of 20 contiguous voxels. All clusters reported *t* > 3.5. Clusters are overlaid on the MNI standard brain with a mask for lobule VII (crus 1, 2, and VIIb) shown in blue and a mask for the vermis shown in light blue. L, Left Hemisphere; R, Right Hemisphere.

An exclusive analysis was performed to determine cerebellar regions specific to memory, language, executive, emotion, and visuospatial scores. Bilateral lobules VII (Crus I–II) and vermis emerged as distinct to memory performance (brown color, Table 4 and Figure 3); the posterior vermis was found to be exclusively involved in language dysfunction (blue color, Table 4 and Figure 3); mild involvement of right lobules VII (Crus I) and left lobule VI was exclusive to executive functioning (pink color, Table 4 and Figure 3); bilateral lobules VI–VII (Crus I) with greater right hemisphere involvement was exclusive to emotion processing (green color, Table 4 and Figure 3), and left lobule VI emerged as exclusive to visuospatial function (red color, Table 4 and Figure 3).

**Table 4:** **Voxel-based morphometry (VBM) findings demonstrating distinct regions of cerebellar gray matter atrophy for memory, language, emotion, executive, and visuospatial performances across all participants (*p* < 0.01) as shown in Figure 3**.

Cerebellar subregions	**Memory**		**Language**		**Executive**		**Emotion**		**Visuospatial**	
	**R**	**L**	**R**	**L**	**R**	**L**	**R**	**L**	**R**	**L**
I–IV										
V										
VI						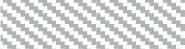		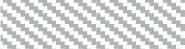		
VII (Crus 1)					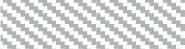			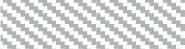		
VII (Crus 2)										
VIIB										
VIII										
IX–X										
Vermis										

*Solid gray color represents areas of substantial correlation, diagonal lines represent areas of milder significance*.

## Discussion

The present study investigated the neural correlates of cognitive and neuropsychiatric performance in the cerebellum of patients with bvFTD. Our results show that cognitive and neuropsychiatric decline across various domains of memory, language, emotion, executive, visuospatial function, and motivation relates to the degree of gray matter degeneration in cerebellar lobules V–VII. Comparisons across task categories were performed to determine overlapping and distinct cerebellar subregions associated with deficits in each cognitive domain. This revealed a convergence across all cognitive and neuropsychiatric substrates on cerebellar lobule VI, with additional associations seen in lobules V and VII (Crus I) for memory, language, executive, and emotion deficits, and a further shared representation present between memory and language dysfunction in the vermis. Most notably, bilateral cerebellar lobules VII and vermis emerged as distinct for deficits in memory processing, the right cerebellar hemisphere associated with emotion dysfunction and the cerebellar vermis was highlighted in language dysfunction.

Although the cerebellum is now known to play a critical role in cognition and neuropsychiatric processes (Schmahmann and Sherman, 1998; Stoodley and Schmahmann, 2010), evidence of its contribution to the cognitive and neuropsychiatric symptoms in bvFTD is only beginning to surface (Tan et al., 2014). The present study demonstrated that degeneration in cerebellar lobules VI–VII impacts on memory, emotion, executive, and visuospatial performances in bvFTD, corroborating findings in healthy humans (Stoodley and Schmahmann, 2009; Keren-Happuch et al., 2014) and the functional topographical organization proposed in the human cerebellum (Krienen and Buckner, 2009; O'Reilly et al., 2010; Stoodley and Schmahmann, 2010; Stoodley et al., 2012). Cerebellar lobules VI–VII have been implicated in language processes in healthy humans and while variable results with regards to whether language function maps predominantly onto the right, left or bilateral cerebellar hemispheres have been found (Krienen and Buckner, 2009; Stoodley and Schmahmann, 2009), we demonstrate here an association between bilateral lobule VI and left Crus I with language dysfunction in bvFTD. We further demonstrated involvement of the posterior vermis, also known as the “limbic cerebellum,” in deficits in the language and memory domains in bvFTD.

Given that intact cognition is multifaceted and contingent upon shared memory, language, executive, emotion, and visuospatial processes, cognitive substrates that each individually also draw upon another, we conducted comparisons across task categories to further identify regions significant to deficits in each domain. Overlap analyses was first performed to determine if within cerebellar subregions implicated across task categories, there existed regions of overlap. Our findings revealed collective clusters within lobules VI and Crus I across deficits in the memory, language, executive, emotion and visuospatial domains, and in the vermis across memory and language dysfunction. We then proceeded to perform comparisons to determine subregions distinct to each category within these cerebellar substructures. Within each of these individual domains, discriminate analysis underscored the bilateral lobules VII and vermis to memory performance, the vermis to language performance, right-lateralised lobules VI and VII (Crus I) to deficits in emotion, and left-lateralized lobule VI to visuospatial dysfunction. Only mild involvement of cerebellar lobules VI and VII (Crus I) was observed with executive dysfunction in the absence of memory, language, emotion and visuospatial substrates. While this may be due to the recruitment of other cognitive domains in the present assessments of executive dysfunction, this is not an isolated report of difficulties in identifying cerebellar regions involved purely in executive function (Stoodley and Schmahmann, 2009).

Intact memory in bvFTD has been found to be contingent upon the integrity of the prefrontal cortices and the limbic “Papez” cortical-subcortical network (Hornberger et al., 2012; Frisch et al., 2013; Irish et al., 2013). Given that connectivity studies have shown closed-loop connections between the Crus with the prefrontal cortices and the vermis with the limbic network (Middleton and Strick, 2001; Kelly and Strick, 2003; O'Reilly et al., 2010), the present findings of distinct lobule VII (Crus) and vermis contributions to memory performance in bvFTD underscores the significance of the cortical-cerebellar network to intact memory processing in bvFTD. Based on the left-hemispheric involvement of the cortical regions in language in bvFTD, we anticipated greater contributions from the right cerebellar hemisphere. However, our results revealed that in the absence of other cognitive task categories, only the vermis emerged as distinct to deficits in language processing in bvFTD, a finding that although not reported in healthy humans (Stoodley and Schmahmann, 2009), is observed in individuals with autism (Riva et al., 2013). Contrary to the expectation that the vermis would be more involved in deficits in emotion processing (Baumann and Mattingley, 2012), predominant right-lateralized lobule VI emerged as distinct with measures of emotion processing, a finding which may relate to the predominant left hemispheric recruitment of various cortices during these tasks in bvFTD (Kumfor et al., 2013; Bertoux et al., 2014).

Apathy is a prominent feature of bvFTD and studies performed so far suggest that the neural basis underlying this lack of motivation is the disruption of the cortical-basal ganglia circuitry in patients with bvFTD (Eslinger et al., 2012; Yi et al., 2013). Despite being a recipient and reciprocal relay of cortical projections via the basal ganglia, cerebellar involvement was not highlighted in these studies. However, one case report demonstrated a simultaneous reduction of apathy and improved metabolism in the bilateral insula and cerebellum with the administration of an N-methyl-D-aspartate antagonist to a patient with bvFTD (Links et al., 2013). Together with the present findings pinpointing right-lateralized cerebellar lobule VI in motivation, it appears that the cerebellum remains largely overlooked in the study of neuropsychiatric deficits in bvFTD, particularly in sporadic disease.

There are several methodological issues that warrant consideration. In order to explore the cerebellar correlates of cognitive and neuropsychiatric measures in this group, covariate analyses was assessed for significance at *p* < 0.01 uncorrected and it was beyond the scope of this study to determine if the cerebellar correlates identified here are specific to bvFTD, or seen across other neurodegenerative diseases as well. However, despite this, it is important to note that our findings here converge well with a growing number of studies that have highlighted cerebellar subregions involved in cognitive and neuropsychiatric measures across cohorts of healthy humans and other lesion models. Finally, cerebellar correlations were not assessed across each bvFTD cohort with controls due to (1) our findings both here (Table 1) and previously (Lillo et al., 2010) of no significant difference in cognitive and neuropsychiatric measures across these two bvFTD cohorts; (2) the substantial body of literature demonstrating that bvFTD and ALSFTD sit on the same disease continuum; (3) sample size (*n* = 15 controls, *n* = 23 bvFTD, *n* = 15 ALSFTD) teamed with the above two rationale and (4) the focus being cerebellar atrophy with cognitive and neuropsychiatric changes rather than motor impairment which, in the case of the later, would warrant analyses to have been performed across each patient group with controls. However, it would be of interest to perform covariation analyses within larger participant cohorts in the future.

In summary, the present findings of significant cerebellar gray matter associations with cognitive and neuropsychiatric processes in bvFTD are consistent with the neural correlates identified with these domains in the cortical regions involved. This emphasizes the involvement of whole brain cortical-cerebellar network in cognition and neuropsychiatric function, highlighting the importance of including cerebellar assessment in whole brain analyses of structural changes in bvFTD.

### Conflict of interest statement

The authors declare that the research was conducted in the absence of any commercial or financial relationships that could be construed as a potential conflict of interest.

## Supplementary material

Supplementary Figure 1 **Voxel-based morphometry findings contrasting gray matter atrophy in bvFTD and ALSFTD in comparison to controls, and bvFTD in comparison to ALSFTD**. Colored voxels show regions that were significant in the analysis for *p* < 0.05 family-wise error (FEW) corrected. Results are published in PLoS ONE 9(8): e105632. doi: 10.1371/journal.pone.0105632 and demonstrate significant cerebellar atrophy in both bvFTD and ALSFTD cohorts in comparison to controls, but no significant difference across patient groups. Clusters are overlaid on the MNI standard brain with a mask for lobule VII (crus 1, 2, and VIIb) shown in blue.

## References

[B1] Andersson J. L. R. Jenkinson M. Smith S. (2007a). Non-linear optimisation FMRIB Technial Report TR07JA1. Oxford: FMRIB Centre.

[B2] Bak T. H. Hodges J. R. (2001). Motor neurone disease, dementia and aphasia: coincidence, co-occurrence or continuum? J. Neurol. 248, 260–270. 10.1007/s004150170199 11374089

[B3] Baumann O. Mattingley J. B. (2012). Functional topography of primary emotion processing in the human cerebellum. Neuroimage 61, 805–811. 10.1016/j.neuroimage.2012.03.044 22465459

[B4] Bertoux M. Volle E. De Souza L. C. Funkiewiez A. Dubois B. Habert M. O. (2014). Neural correlates of the mini-SEA (social cognition and emotional assessment) in behavioral variant frontotemporal dementia. Brain Imaging Behav. 8, 1–6. 10.1007/s11682-013-9261-0 24078043

[B5] Brooks B. R. Miller R. G. Swash M. Munsat T. L. (2000). El Escorial revisited: revised criteria for the diagnosis of amyotrophic lateral sclerosis. Amyotroph. Lateral Scler. Other Motor Neuron Disord. 1, 293–299. 10.1080/146608200300079536 11464847

[B6] Eslinger P. J. Moore P. Antani S. Anderson C. Grossman M. (2012). Apathy in frontotemporal dementia: behavioral and neuroimaging correlates. Behav. Neurol. 25, 127–136. 10.1155/2012/286427 22425723 PMC3640327

[B7] Frisch S. Dukart J. Vogt B. Horstmann A. Becker G. Villringer A.. (2013). Dissociating memory networks in early Alzheimer's disease and frontotemporal lobar degeneration - a combined study of hypometabolism and atrophy. PLoS ONE 8:e55251. 10.1371/journal.pone.0055251 23457466 PMC3573064

[B8] Goodglass H., K. E. (2000). Boston Naming Test, 2nd Edn. Philadelphia, PA: Lippincott Williams & Wilkins.

[B9] Grossman M. McMillan C. Moore P. Ding L. Glosser G. Work M.. (2004). What's in a name: voxel-based morphometric analyses of MRI and naming difficulty in Alzheimer's disease, frontotemporal dementia and corticobasal degeneration. Brain 127, 628–649. 10.1093/brain/awh075 14761903

[B10] Hornberger M. Piguet O. Kipps C. Hodges J. R. (2008). Executive function in progressive and nonprogressive behavioral variant frontotemporal dementia. Neurology 71, 1481–1488. 10.1212/01.wnl.0000334299.72023.c8 18981369

[B11] Hornberger M. Wong S. Tan R. Irish M. Piguet O. Kril J.. (2012). *In vivo* and post-mortem memory circuit integrity in frontotemporal dementia and Alzheimer's disease. Brain 135, 3015–3025. 10.1093/brain/aws239 23012333

[B12] Huey E. D. Goveia E. N. Paviol S. Pardini M. Krueger F. Zamboni G.. (2009). Executive dysfunction in frontotemporal dementia and corticobasal syndrome. Neurology 72, 453–459. 10.1212/01.wnl.0000341781.39164.26 19188577 PMC2677529

[B13] Irish M. Devenney E. Wong S. Dobson-Stone C. Kwok J. B. Piguet O.. (2013). Neural substrates of episodic memory dysfunction in behavioural variant frontotemporal dementia with and without C9ORF72 expansions. Neuroimage Clin. 2, 836–843. 10.1016/j.nicl.2013.06.005 24179835 PMC3778250

[B14] Irish M. Piguet O. Hodges J. R. Hornberger M. (2014). Common and unique gray matter correlates of episodic memory dysfunction in frontotemporal dementia and Alzheimer's Disease. Hum. Brain Mapp. 35, 1422–1435. 10.1002/hbm.22263 23670951 PMC6869668

[B15] Kelly R. M. Strick P. L. (2003). Cerebellar loops with motor cortex and prefrontal cortex of a nonhuman primate. J. Neurosci. 23, 8432–8444. 12968006 10.1523/JNEUROSCI.23-23-08432.2003 PMC6740694

[B16] Keren-Happuch E. Chen S. H. A. Ho M. H. R. Desmond J. E. (2014). A meta-analysis of cerebellar contributions to higher cognition from PET and fMRI Studies. Hum. Brain Mapp. 35, 593–615. 10.1002/hbm.22194 23125108 PMC3866223

[B17] Krienen F. M. Buckner R. L. (2009). Segregated fronto-cerebellar circuits revealed by intrinsic functional connectivity. Cereb. Cortex 19, 2485–2497. 10.1093/cercor/bhp135 19592571 PMC2742600

[B18] Kumfor F. Irish M. Hodges J. R. Piguet O. (2013). Discrete neural correlates for the recognition of negative emotions: insights from frontotemporal dementia. PLoS ONE 8:e67457. 10.1371/journal.pone.0067457 23805313 PMC3689735

[B19] Kumfor F. Sapey-Triomphe L. A. Leyton C. E. Burrell J. R. Hodges J. R. Piguet O. (2014). Degradation of emotion processing ability in corticobasal syndrome and Alzheimer's disease. Brain 137, 3061–3072. 10.1093/brain/awu246 25227744

[B20] Lillo P. Garcin B. Hornberger M. Bak T. H. Hodges J. R. (2010). Neurobehavioral features in frontotemporal dementia with amyotrophic lateral sclerosis. Arch. Neurol. 67, 826–830. 10.1001/archneurol.2010.146 20625088

[B21] Links K. A. Black S. E. Graff-Guerrero A. Wilson A. A. Houle S. Pollock B. G.. (2013). A case of apathy due to frontotemporal dementia responsive to memantine. Neurocase 19, 256–261. 10.1080/13554794.2012.667120 22515731

[B22] Middleton F. A. Strick P. L. (2000). Basal ganglia and cerebellar loops: motor and cognitive circuits. Brain Res. Brain Res. Rev. 31, 236–250. 10.1016/S0165-0173(99)00040-5 10719151

[B23] Middleton F. A. Strick P. L. (2001). Cerebellar projections to the prefrontal cortex of the primate. J. Neurosci. 21, 700–712. 11160449 10.1523/JNEUROSCI.21-02-00700.2001 PMC6763818

[B24] Miller L. A. Hsieh S. Lah S. Savage S. Hodges J. R. Piguet O. (2012). One size does not fit all: face emotion processing impairments in semantic dementia, behavioural-variant frontotemporal dementia and Alzheimer's disease are mediated by distinct cognitive deficits. Behav. Neurol. 25, 53–60. 10.1155/2012/683052 22207423 PMC5294238

[B25] Mioshi E. Dawson K. Mitchell J. Arnold R. Hodges J. R. (2006). The Addenbrooke's Cognitive Examination Revised (ACE-R): a brief cognitive test battery for dementia screening. Int. J. Geriatr. Psychiatry 21, 1078–1085. 10.1002/gps.1610 16977673

[B26] Nichols T. E. Holmes A. P. (2002). Nonparametric permutation tests for functional neuroimaging: a primer with examples. Hum. Brain Mapp. 15, 1–25. 10.1002/hbm.1058 11747097 PMC6871862

[B27] O'Reilly J. X. Beckmann C. F. Tomassini V. Ramnani N. Johansen-Berg H. (2010). Distinct and overlapping functional zones in the cerebellum defined by resting state functional connectivity. Cereb. Cortex 20, 953–965. 10.1093/cercor/bhp157 19684249 PMC2837094

[B28] Pennington C. Hodges J. R. Hornberger M. (2011). Neural correlates of episodic memory in behavioral variant frontotemporal dementia. J. Alzheimers Dis. 24, 261–268. 10.3233/JAD-2011-101668 21239854

[B29] Rabinovici G. D. Seeley W. W. Kim E. J. Gorno-Tempini M. L. Rascovsky K. Pagliaro T. A.. (2007). Distinct MRI atrophy patterns in autopsy-proven Alzheimer's disease and frontotemporal lobar degeneration. Am. J. Alzheimers. Dis. Other Demen. 22, 474–488. 10.1177/1533317507308779 18166607 PMC2443731

[B30] Raczka K. A. Becker G. Seese A. Frisch S. Heiner S. Marschhauser A.. (2010). Executive and behavioral deficits share common neural substrates in frontotemporal lobar degeneration - A pilot FDG-PET study. Psychiatry Res. 182, 274–280. 10.1016/j.pscychresns.2010.02.009 20493673

[B31] Rascovsky K. Hodges J. R. Knopman D. Mendez M. F. Kramer J. H. Neuhaus J.. (2011). Sensitivity of revised diagnostic criteria for the behavioural variant of frontotemporal dementia. Brain 134, 2456–2477. 10.1093/brain/awr179 21810890 PMC3170532

[B32] Riva D. Annunziata S. Contarino V. Erbetta A. Aquino D. Bulgheroni S. (2013). Gray matter reduction in the vermis and CRUS-II is associated with social and interaction deficits in low-functioning children with autistic spectrum disorders: a VBM-DARTEL study. Cerebellum 12, 676–685. 10.1007/s12311-013-0469-8 23572290

[B33] Savage S. Hsieh S. Leslie F. Foxe D. Piguet O. Hodges J. R. (2013). Distinguishing subtypes in primary progressive aphasia: application of the Sydney language battery. Dement. Geriatr. Cogn. Disord. 35, 208–218. 10.1159/000346389 23467307

[B34] Schmahmann J. D. Sherman J. C. (1998). The cerebellar cognitive affective syndrome. Brain 121, 561–579. 10.1093/brain/121.4.561 9577385

[B35] Seeley W. W. Crawford R. Rascovsky K. Kramer J. H. Weiner M. Miller B. L.. (2008). Frontal paralimbic network atrophy in very mild behavioral variant frontotemporal dementia. Arch. Neurol. 65, 249–E241. 10.1001/archneurol.2007.38 18268196 PMC2544627

[B36] Smith S. M. (2002). Fast robust automated brain extraction. Hum. Brain Mapp. 17, 143–155. 10.1002/hbm.10062 12391568 PMC6871816

[B37] Smith S. M. Nichols T. E. (2009). Threshold-free cluster enhancement: addressing problems of smoothing, threshold dependence and localisation in cluster inference. Neuroimage 44, 83–98. 10.1016/j.neuroimage.2008.03.061 18501637

[B38] Stoodley C. J. Schmahmann J. D. (2009). Functional topography in the human cerebellum: a meta-analysis of neuroimaging studies. Neuroimage 44, 489–501. 10.1016/j.neuroimage.2008.08.039 18835452

[B39] Stoodley C. J. Schmahmann J. D. (2010). Evidence for topographic organization in the cerebellum of motor control versus cognitive and affective processing. Cortex 46, 831–844. 10.1016/j.cortex.2009.11.008 20152963 PMC2873095

[B40] Stoodley C. J. Valera E. M. Schmahmann J. D. (2012). Functional topography of the cerebellum for motor and cognitive tasks: an fMRI study. Neuroimage 59, 1560–1570. 10.1016/j.neuroimage.2011.08.065 21907811 PMC3230671

[B41] Tan R. H. Devenney E. Dobson-Stone C. Kwok J. B. Hodges J. R. Kiernan M. C.. (2014). Cerebellar integrity in the amyotrophic lateral sclerosis - frontotemporal dementia continuum. PLoS ONE 9:e105632. 10.1371/journal.pone.0105632 25144223 PMC4140802

[B42] Wedderburn C. Wear H. Brown J. Mason S. J. Barker R. A. Hodges J.. (2008). The utility of the Cambridge Behavioural Inventory in neurodegenerative disease. J. Neurol. Neurosurg. Psychiatr. 79, 500–503. 10.1136/jnnp.2007.122028 17846114

[B43] Woost T. B. Dukart J. Frisch S. Barthel H. Sabri O. Mueller K.. (2013). Neural correlates of the DemTect in Alzheimer's disease and frontotemporal lobar degeneration - A combined MRI and FDG-PET study. Neuroimage Clin. 2, 746–758. 10.1016/j.nicl.2013.05.008 24179826 PMC3777755

[B44] Yi D. S. Bertoux M. Mioshi E. Hodges J. Hornberger M. (2013). Fronto-striatal atrophy correlates of neuropsychiatric dysfunction in frontotemporal dementia (FTD) and Alzheimer's disease (AD). Dement Neuropsychol. 7, 75–82. 10.1590/S1980-57642013DN70100012 PMC5619548 29213823

[B45] Young Aw P. D. Calder A. J. Sprengelmeyer R. Ekman P. (2002). Facial Expressions of Emotion - Stimuli and Tests (FEEST). Bury St Edmunds: Thames Valley Test Company.

[B46] Zhang Y. Brady M. Smith S. (2001). Segmentation of brain MR images through a hidden Markov random field model and the expectation-maximization algorithm. IEEE Trans. Med. Imaging 20, 45–57. 10.1109/42.906424 11293691

