# Supplementary figures and images for: Terra incognita—cerebellar contributions to neuropsychiatric and cognitive dysfunction in behavioral variant frontotemporal dementia
Source: Front Aging Neurosci. 2015 Jul 2;7:121. doi: 10.3389/fnagi.2015.00121 (PMC4488961; doi:10.3389/fnagi.2015.00121)

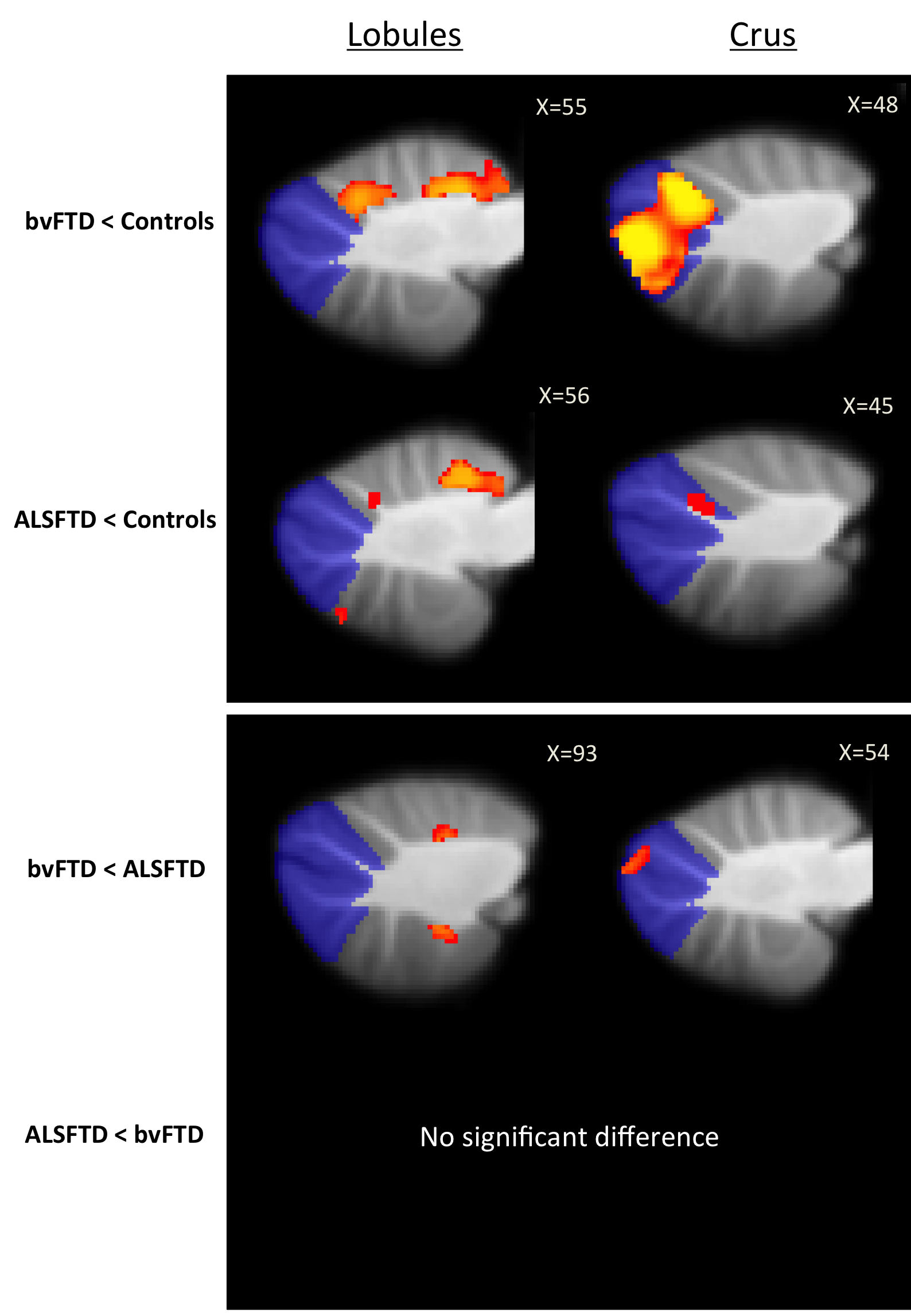

Supplement: Supplementary Figure 1 — Voxel-based morphometry findings contrasting gray matter atrophy in bvFTD and ALSFTD in comparison to controls, and bvFTD in comparison to ALSFTD. Colored voxels show regions that were significant in the analysis for p < 0.05 family-wise error (FEW) corrected. Results are published in PLoS ONE 9(8): e105632. doi: 10.1371/journal.pone.0105632 and demonstrate significant cerebellar atrophy in both bvFTD and ALSFTD cohorts in comparison to controls, but no significant difference across patient groups. Clusters are overlaid on the MNI standard brain with a mask for lobule VII (crus 1, 2, and VIIb) shown in blue. [file Image1.TIF]
